# AI-Generated Podcasts as a Supplemental Pharmacology Learning Tool: A Feasibility Study in Undergraduate Medical Education

**DOI:** 10.1007/s40670-025-02521-6

**Published:** 2025-10-02

**Authors:** Keshab Raj Paudel

**Affiliations:** Department of Biomedical Sciences, Burrell College of Osteopathic Medicine, Melbourne, FL USA

**Keywords:** Artificial intelligence, Asynchronous learning, Medical education, Pharmacology podcasts

## Abstract

Pharmacology remains a cognitively demanding discipline for medical students due to its volume and integration of complex pathophysiological principles. Educational podcasts have emerged as accessible, asynchronous learning resources in health professions education. With recent advances in artificial intelligence (AI), automated guided-content generation provides new avenues for educational innovation. This study evaluates the feasibility, utilization, and learner reception of AI-generated pharmacology podcasts in undergraduate medical education at a US osteopathic medical school. In a retrospective observational study, pharmacology lectures across four organ system–based courses were converted into AI-generated podcast episodes using the NotebookLM and Riverside.fm platforms. Podcasts were made available via the Leo Learning Management System. Download metrics, presence of embedded practice questions, and qualitative student feedback were analyzed. Ten AI-generated podcasts were deployed across first- and second-year osteopathic medical courses. The average duration of the podcasts was 18.1 min. The range for AI podcast downloads was 6–25% whereas it was 50–97% for lecture slides and 1–9% for recommended pharmacology textbooks. Topics such as gastrointestinal pharmacology and Parkinson disease had the highest engagement. Podcasts with integrated practice questions showed slightly increased engagement. Students valued the format for its portability, clarity, and reinforcement of lecture content. AI-generated podcasts represent a scalable, portable, and positively received modality for pharmacology instruction. This study demonstrates early feasibility and acceptance of AI tools in augmenting traditional didactics. Further research is needed to assess impacts on learning outcomes and long-term retention.

## Introduction

Pharmacology is a critical and content-dense domain within medical education. Its heavy reliance on memorization, coupled with the breadth and depth of pharmacokinetic and pharmacodynamic principles, poses challenges for learners at various stages of their training [1]. Students frequently report pharmacology as among the most difficult subjects in the preclinical curriculum due to time constraints, volume overload, and lack of clinical context [2, 3].

The emergence of educational podcasts has introduced new methods to deliver content in flexible and accessible formats. Podcasts support on-demand, mobile learning that complements traditional lectures and textbooks [4, 5]. Multiple studies suggest that podcasts can enhance learner engagement, improve comprehension, and increase satisfaction, particularly in content-heavy courses [6, 7].

Simultaneously, advances in generative AI have expanded the capacity to create customized educational materials. AI platforms such as Google’s NotebookLM and OpenAI’s GPT models enable rapid generation of didactic content in conversational formats. These outputs can be easily converted into podcasts using user-friendly platforms like Riverside.fm. Such workflows are potentially scalable, cost-effective, and time-efficient, making them suitable for institutions with limited faculty resources [8, 9].

However, limited research has assessed the practical use of AI-generated podcasts in health professions education. This study explores their implementation in pharmacology instruction for first- and second-year osteopathic medical students at a US medical school, evaluating their feasibility, patterns of usage, and student perceptions.

Objectives of the study were to assess the feasibility of producing and distributing AI-generated pharmacology podcasts, to analyze patterns of podcast utilization by medical students, to collect and interpret student feedback on podcast content and delivery, and to explore the potential of AI podcasts as a supplemental learning resource in pharmacology.

## Methods

### Study Design

This was a retrospective observational study conducted at a single US osteopathic medical school. AI-generated podcasts were implemented across four courses: Nervous System II (NVSII), Behavioral Medicine and Psychiatry (BMP), Gastrointestinal System I (GISI), and Pathological Basis of Disease (PBD). The system courses that involved pharmacology were Molecules to People, Cardiovascular, Respiratory, Gastrointestinal, Reproductive, Endocrine, Renal, Nervous, Behavioral Medicine and Psychiatry, Musculoskeletal, Pathological Basis of Disease, and Pathologic Overview of Medicine, and these system courses were distributed across year 1 and year 2 curriculum. The podcasts were made as supplemental learning resources for the four courses with ten pharmacology topics as the instructor (researcher) introduced the AI podcast as an innovative supplemental instructional method in the courses the instructor was involved in. The study was conducted from January to May 2025.

### Podcast Development

Pharmacology lecture slides were converted to PDFs and uploaded to Google’s NotebookLM platform. Using the “deep dive conversation” tool, customized dialogues between a faculty expert and a student host were generated. Specific instructional directives were given to the AI to include the name of the instructor as resource person, focus on highlighted areas on the PDF, and include practice questions with explanations from the PDF generated from lecture slides. Each script was edited for content accuracy and duration of podcast using the Riverside.fm platform. Post-production ensured optimal audio quality and consistency. Podcasts were 11–30 min in length (mean, 18.1 min).

### Implementation

The podcasts were uploaded along with lecture slides and links to recommended Pharmacology textbooks to the Leo Learning Management System (LMS) and labeled as “AI audio podcast.” In a few cases, separate files of practice questions were also uploaded (for details, refer to Table 1). Students were informed of their availability through email and lecture announcements. The delivery modality of the accompanying lectures varied (in-person or pre-recorded).

**Table 1:** Characteristics of pharmacology topics and resources delivered to the medical students

Course, no. of students (*N*)	Topic	Resources	Downloads	%	Delivery
NVSII *N* = 175	Parkinson Disease Pharmacotherapy	Lecture slides	168	96.0	Pre-recorded
		Practice questions	113	64.6	
		AI pod cast without practice questions	25	14.3	
		Link to Katzung’s Basic & Clinical Pharmacology, 16 ed	3	1.7	
		Link to Examination and Board Review, 14 ed	3	1.7	
		Link to Goodman & Gilman, 14 ed	3	1.7	
	Pharmacology of Multiple Sclerosis	Lecture slides with embedded practice questions with no answer	103	58.9	In-person
		Lecture slides with embedded practice questions with answer	136	77.7	
		AI podcast with practice questions	21	12.0	
		Link to Harrison’ Principle of Internal Medicine, 21 ed	4	2.3	
	Headache Pharmacology	Lecture slides with embedded practice questions	165	94.3	Pre-recorded
		AI podcast with practice questions	21	12.0	
		Link to Examination and Board Review, 14 ed	10	5.7	
		Link to Katzung’s Basic & Clinical Pharmacology, 16 ed	1	0.6	
		Link to Goodman & Gilman, 14 ed	0	0.0	
	Neurodegenerative Disease Pharmacology	Lecture slides with embedded practice questions	163	93.1	Pre-recorded
		AI podcast with practice questions	19	10.9	
		Link to Katzung’s Basic & Clinical Pharmacology, 16 ed	2	1.1	
		Link to Goodman & Gilman, 14 ed	1	0.6	
		Lecture slides with embedded practice questions with answer	144	82.3	
	Drugs for Spasms and Spasticity	Lecture slides with embedded practice questions with no answer	87	49.7	In-person
		AI podcast with practice questions	10	5.7	
		Link to Examination and Board Review, 14 ed	9	5.1	
		Link to Katzung’s Basic & Clinical Pharmacology, 16 ed	2	1.1	
		Link to Goodman & Gilman, 14 ed	1	0.6	
	Opioid Pharmacology	Lecture slides	170	97.1	Pre-recorded
		Practice questions	133	76.0	
		AI podcast	20	11.4	
		Link to Examination and Board Review, 14 ed	5	2.9	
		Link to Katzung’s Basic & Clinical Pharmacology, 16 ed	1	0.6	
		Link to Goodman & Gilman, 14 ed	1	0.6	
	Pharmacological Management of ADHD and Narcolepsy	Lecture slides with embedded practice questions	169	96.6	Pre-recorded
		AI podcast with practice questions	18	10.3	
		Link to Examination and Board Review, 14 ed	9	5.1	
		Link to Katzung’s Basic & Clinical Pharmacology, 16 ed	2	1.1	
		Link to Goodman & Gilman, 14 ed	1	0.6	
BMP *N* = 175	Pharmacology of Opioid and Stimulant Abuse	Lecture slides	168	96.0	Pre-recorded
		Practice questions	127	72.6	
		AI podcast	15	8.6	
		Link to Examination and Board Review, 14 ed	6	3.4	
		Link to Katzung’s Basic & Clinical Pharmacology, 16 ed	1	0.6	
		Link to Goodman & Gilman, 14 ed	1	0.6	
GISI *N* = 299	Pharmacological Regulation of GI Autonomic Function	Lecture slides with embedded practice questions with no answer	228	76.5	In-person
		Lecture slides with embedded practice questions with answer	191	64.1	
		AI podcast with practice questions	75	25.2	
		Link to Katzung’s Basic & Clinical Pharmacology, 16 ed	14	4.7	
		Link to Examination and Board Review, 14 ed	27	9.1	
PBD *N* = 299	Introduction to Toxicology	Lecture slides with embedded practice questions with answer	221	73.9	In-person
		Lecture slides with embedded practice questions with no answer	177	59.2	
		Practice questions (different)	67	22.4	
		AI podcast with practice questions	40	13.4	
		Link to Examination and Board Review, 14 ed	15	5.0	
		Link to Katzung’s Basic & Clinical Pharmacology, 16 ed	7	2.3	

### Data Collection

The following data were collected: download statistics for lecture slides, AI podcasts, and recommended Pharmacology textbooks, and podcast topic and duration, course and number of enrolled students, number of downloads and percentage engagement, presence or absence of practice questions, mode of primary instruction (pre-recorded vs. in-person), and qualitative feedback from regular course evaluations.

## Results

Ten AI-generated pharmacology podcasts were developed and deployed across four systems-based courses. Table 1 details podcast characteristics, resources used, download statistics, and delivery methods. Tables 1, 2, and 3 summarize key metrics. *Highest engagement*: The GI Autonomic Regulation podcast had 75 downloads (25.2% engagement rate). *Practice questions*: Podcasts that included practice questions had a slightly higher mean download rate (13.1%) than those without (11.4%) (Table 3). *Course formats*: Podcasts accompanying in-person lectures showed slightly higher engagement (average 14.1%) than those paired with pre-recorded lectures (11.3%). *Most accessed topics*: Pharmacological Regulation of GI Autonomic Function (75 downloads), Parkinson Disease (25 downloads), Introduction to Toxicology (40 downloads), and ADHD/Narcolepsy (18 downloads).

**Table 2:** Download statistics of pharmacology AI podcast

AI podcast	Duration of podcast	Practice questions included	No. of downloads	% of download	Mode of instruction
Parkinson Disease Pharmacotherapy	11.44	No	25	14.3	Pre-recorded
Pharmacology of Multiple Sclerosis	24.22	Yes	21	12.0	In-person
Headache Pharmacology	16.01	Yes	21	12.0	Pre-recorded
Neurodegenerative Disease Pharmacology	18	Yes	19	10.9	Pre-recorded
Drugs for Spasms and Spasticity	14.08	Yes	10	5.7	In-person
Opioid Pharmacology	20.39	No	20	11.4	Pre-recorded
Pharmacological Management of ADHD and Narcolepsy	20.53	Yes	18	10.3	Pre-recorded
Pharmacology of Opioid and Stimulant Abuse	14.32	No	15	8.6	Pre-recorded
Pharmacological Regulation of GI Autonomic Function	11.36	Yes	75	25.2	In-person
Introduction to Toxicology	30.52	Yes	40	13.4	In-person
Mean	18.09		23.30	12.36	
SD	5.46		9.65	4.60

**Table 3:** Download statistics of pharmacology AI podcast with and without practice questions and for in-person and pre-recorded lectures

AI podcast with practice questions		AI podcast without practice questions		AI podcast for in-person lectures		AI podcast for pre-recorded lectures	
No. of downloads *Mean* ± *SD* *(29* ± *21*)	% of download *Mean* ± *SD* *(13.1* ± *5.2)*	No. of downloads *Mean* ± *SD* *(20* ± *4*)	% of download *Mean* ± *SD* *(11.4* ± *2.3*)	No. of downloads *Mean* ± *SD* *(37* ± *29*)	% of download *Mean* ± *SD* *(14.1* ± *7.0*)	No. of downloads *Mean* ± *SD* *(20* ± *3*)	% of download *Mean* ± *SD* *(11.3* ± *1.6*)
21	14.3	25	14.3	21	12	25	14.3
21	12	20	11.4	10	5.7	21	12
19	10.9	15	8.6	75	25.2	19	10.9
10	5.7	-	-	40	13.4	20	11.3
18	10.3	-	-	-	-	18	10.3
75	25.2	-	-	-	-	15	8.6
40	13.4	-	-	-	-	-	-

Student Feedback: Comments received in regular teaching evaluations included: “*The AI podcast was helpful and a great pairing with your lecture.*” “*I appreciated this! It allowed repetition while I completed daily tasks.*” “*Helpful first pass at the material, and convenient for listening while driving.*” “*Podcast was interesting and something to quickly summarize after studying*.” This positive tone of students’ feedback supports podcast acceptability and relevance.

## Discussion

This study highlights the practical application and initial reception of AI-generated podcasts as a supplemental learning resource in undergraduate pharmacology education. Pharmacology, as a foundational science in medical curricula, demands cognitive endurance, substantial memorization—for example drugs’ names, and the integration of mechanistic and clinical knowledge. Traditional lecture-based formats, while efficient for information delivery, often fail to meet the diverse learning preferences and cognitive needs of modern learners [3]. Our findings suggest that AI-generated podcasts—featuring concise, conversational, and clinically integrated content—are both feasible to implement and well-received by students, offering a novel strategy to enhance pharmacology learning.

Consistent with prior literature, the positive student feedback we observed affirms the educational value of podcasts in health professions education. Learners appreciated the flexibility, portability, and reinforcement afforded by podcast episodes, echoing results from studies that associate podcasts with improved learner satisfaction, engagement, and perceived utility [4–7]. In particular, the ability to review content while multitasking (e.g., commuting, exercising) reflects an emerging preference among adult learners for mobile and asynchronous educational tools [10, 11]. This aligns with self-determination theory, which emphasizes autonomy and flexibility as critical drivers of learner motivation [12].

From a theoretical perspective, our approach is supported by Mayer’s cognitive theory of multimedia learning, which advocates multisensory input to optimize memory retention and transfer [13]. Podcasts leverage auditory channels and allow learners to process information in manageable segments, potentially mitigating cognitive overload. This is particularly pertinent in pharmacology, where the quantity and complexity of information often contribute to learner burnout and disengagement [3]. Furthermore, the deliberate integration of clinical-style questions within each episode likely promotes active processing, consistent with constructivist theory and the generation effect, both of which enhance learning through retrieval and elaboration [14, 15].

The novelty of our work lies not only in the podcast format but in the method of content generation. By utilizing a generative AI platform (NotebookLM), we streamlined the scripting process through automated summarization and dialogue generation, significantly reducing the time and effort traditionally required for multimedia content development. This model of instructional design is particularly advantageous for faculty in resource-constrained environments where teaching loads and administrative responsibilities limit opportunities for content creation [8, 9]. Our findings support the scalability of this approach: once a framework for AI-assisted podcast production is established, content can be rapidly replicated, revised, and tailored to fit different topics, courses, or learner levels.

Interestingly, while absolute download numbers varied, there was some utilization across all podcast topics, including for those in-person lectures which included practice questions (Table 1). This finding suggests that the perceived utility of AI-generated podcasts may extend across all topics and subjects even if they are taught in-person with a focus on important topics with the help of clinical cases (practice questions). Notably, podcasts with embedded practice questions demonstrated slightly higher engagement than those without, indicating a possible additive value of interactive elements (Tables 2 and 3). These findings parallel previous work demonstrating that educational podcasts are most effective when they stimulate active engagement, such as through quizzes, case-based discussions, or reflection prompts [16, 17].

Despite promising signs of feasibility and positive reception, several limitations should temper interpretation. First, engagement was measured through download counts, which do not confirm whether students listened to the entire episode or integrated the material into their learning strategies. Future studies should incorporate in-app analytics or learner-reported behavior to assess listening fidelity and patterns [18]. Second, while qualitative feedback was overwhelmingly positive, it was collected via routine evaluations and not systematically analyzed using validated thematic analysis methods. Structured qualitative methods and focus group interviews would yield richer insights into the learner’s experience and potential improvements.

Moreover, the impact of AI-generated podcasts on academic performance, knowledge retention, or long-term comprehension was not evaluated in this study. As a result, we cannot claim efficacy beyond subjective learner perception. Future investigations should adopt experimental or quasi-experimental designs comparing podcast use to conventional study tools or measuring performance on pre- and post-tests [19]. It is also worth exploring how students integrate podcasts into their broader learning ecology, whether they serve as primary resources, review tools, or substitutes for attendance and reading.

Importantly, the integration of generative AI into educational content raises questions around content accuracy, academic integrity, and the evolving role of faculty. While faculty oversight was used in this study to review and edit AI-generated scripts, unchecked reliance on AI could propagate inaccuracies or omit clinically relevant nuance. Responsible use of AI in medical education must include rigorous content validation and transparency about authorship and editorial control [20]. Educators must also be trained not just in AI tools, but in digital pedagogy—how to align emerging technologies with evidence-based teaching principles [21].

Lastly, while the scope of this study was limited to pharmacology, the method has broader implications. Other preclinical disciplines—such as microbiology, physiology, or pathology—may benefit from similar AI-assisted podcast development, particularly in systems-based curricula. As health professions education increasingly embraces hybrid and flipped-classroom models, the demand for high-quality, low-cost, and flexible educational tools will continue to grow [22, 23]. AI-generated podcasts, if carefully designed and quality assured, represent a promising addition to this evolving educational toolkit. However, it is important to note that these podcasts are supplemental materials and are not a replacement for in-person pedagogy. Additionally, AI tools may have beneficial effects in terms of efficiency and accessibility. However, these tools may reduce critical thinking and peer interaction in health professional education [24].

In summary, this study contributes to the growing body of literature on digital learning innovations by demonstrating that AI-generated podcasts are not only technically feasible but are favorably received by learners in a challenging foundational course. As technology continues to transform how we teach and learn, the integration of generative AI into pedagogical practices—when guided by sound instructional design and faculty oversight—may enhance accessibility, engagement, and educational outcomes in medical education.

Limitations of the study are as follows: download numbers do not confirm full listening or content mastery, or same students getting engaged in downloading and listening to the podcasts, and the study is confined to a single institution and a specific group of pharmacology lectures.

## Conclusion

AI-generated podcasts represent a feasible and innovative tool for supplemental pharmacology education. Their scalability, low cost, and adaptability make them promising additions to medical curricula, especially in content-heavy disciplines. Future studies should use controlled designs to assess their impact on knowledge retention, exam performance, and long-term application of pharmacological principles.
